# Body Mass Index and Disease Activity Are Associated With Moderate to Severe Disability in Crohn's Disease: A Cross-Sectional Study in Shanghai

**DOI:** 10.3389/fmed.2021.662488

**Published:** 2021-07-09

**Authors:** DongSheng Bian, Yongmei Jiang, Yubei Gu, Zirui He, Qi Chen, Yonghua Tang, Jie Zhong, Yongmei Shi

**Affiliations:** ^1^Department of Clinical Nutrition, School of Medicine, Ruijin Hospital Affiliated to Shanghai Jiao Tong University, Shanghai, China; ^2^Department of Gastroenterology, School of Medicine, Ruijin Hospital Affiliated to Shanghai Jiao Tong University, Shanghai, China; ^3^Department of Gastrointestinal Surgery, School of Medicine, Ruijin Hospital Affiliated to Shanghai Jiao Tong University, Shanghai, China; ^4^Department of Radiology, School of Medicine, Ruijin Hospital Affiliated to Shanghai Jiao Tong University, Shanghai, China

**Keywords:** disability, Crohn's disease, risk factors, disease activity, sarcopenia, body mass index

## Abstract

**Background:** The inflammatory bowel disease disability index (IBD-DI) was used to access body functional consequences and disease burden. However, Chinese population data are considerably limited.

**Objective:** We aimed to screen for disability in patients with Crohn's disease (CD) and to assess potential associations with clinical parameters as well as indices related to sarcopenia.

**Methods:** This cross-sectional study includes 146 CD patients from Ruijin Hospital in Shanghai, China. All patients were screened for disability and sarcopenia on the basis of the IBD-DI scale, and the criteria for Asian Working Group for Sarcopenia, respectively. Clinical and demographic variables were collected.

**Results:** Approximately 52.05% of the subjects suffered from moderate or severe disabilities. The prevalence of sarcopenia (48.68 vs. 31.43%, *P* = 0.043), Patient-Generated Subjective Global Assessment score or PG-SGA≥4 (39.47 vs. 17.14%, *P* = 0.003), and high-level C- reactive protein (27.63 vs. 11.43%, *P* = 0.021) were higher in patients with moderate to severe disability than in those without to minimal disability. By multivariate regression modeling, the following were identified as independent factors related to moderate to severe disability: disease activity (OR:10.47, 95% CI: 2.09–52.42) and body mass index (BMI) (OR:4.11, 95% CI: 1.80–9.38).

**Conclusions:** Disability is common in CD patients. Our study showed that moderate to severe disability is not directly associated with muscle mass or muscle quantity but is mostly correlated with disease activity as well as BMI. Thus, close monitoring and follow-up should be conducted on patients who are at high risk of disability, and effective measures should be taken, which may be the best way to prevent disability.

## Introduction

The prevalence of inflammatory bowel disease (IBD) has markedly increased in mainland China (1). Crohn's disease (CD) is a chronic inflammatory bowel disorder that may affect any part of the digestive tract, causing abdominal pain and diarrhea, subsequently leading to functional disability and life-threatening complications (2). CD patients are characterized by their lifelong disease, marked by episodes of remission and relapse (3). Thus, living with CD may negatively affect the physical, psychological, social, and familial quality of life (4–6). Numerous disease-related questionnaires have been developed to evaluate patient-reported outcomes in CD (7–10). Nonetheless, the majority of CD studies focus on health-related quality of life (such as the inflammatory bowel disease questionnaire) assessments, a subjective assessment about the limitations caused by the disease with high uncertainty. Disability assessment determines function loss in an individual and the social cost of CD.

The International Classification of Functioning, Disability, and Health (ICF) of the World Health Organization defines disability as an objective measure of loss of functioning and impairment in patient activity (11). The Inflammatory Bowel Disease Disability Index (IBD-DI) was developed (12) and updated in 2017 (13). The IBD-DI includes body functions, body structures, as well as activity and participation. Studies confirmed that the IBD-DI was associated with gender, clinical disease activity, and disease duration in the West (14–16). However, our literature review revealed that studies are rarely conducted on conditions and clinical results related to disability in IBD patients. This situation has been exacerbated by the allocation of greater funding for basic research instead of clinical studies. Moreover, in CD, risk factors, disease features, economic conditions, cultural background, and medical systems do not completely overlap between the East and the West. Therefore, more research on disability needs to be conducted on the Chinese population.

Inflammatory bowel diseases are often associated with malnutrition and significant alteration in body composition (17). Bryant et al. found that muscle depletion among IBD patients may affect the quality of life (18). Norman et al. suggested that malnutrition-related factors can reduce the quality of life in IBD patients (19). None of the available studies assessed the relationship between disability and body composition.

Liu et al. (20) reported that a large proportion of Chinese IBD patients experience impaired health-related quality of life. Nonetheless, considerably few studies have focused on disability and risk factors in Chinese CD patients. Thus, this study aimed to evaluate disability prevalence among CD patients, evaluated the correlation between disability and body composition, as well as the risk factors that may be associated with a CD-related disability.

## Materials and Methods

### Study Participants

**Figure 1:**
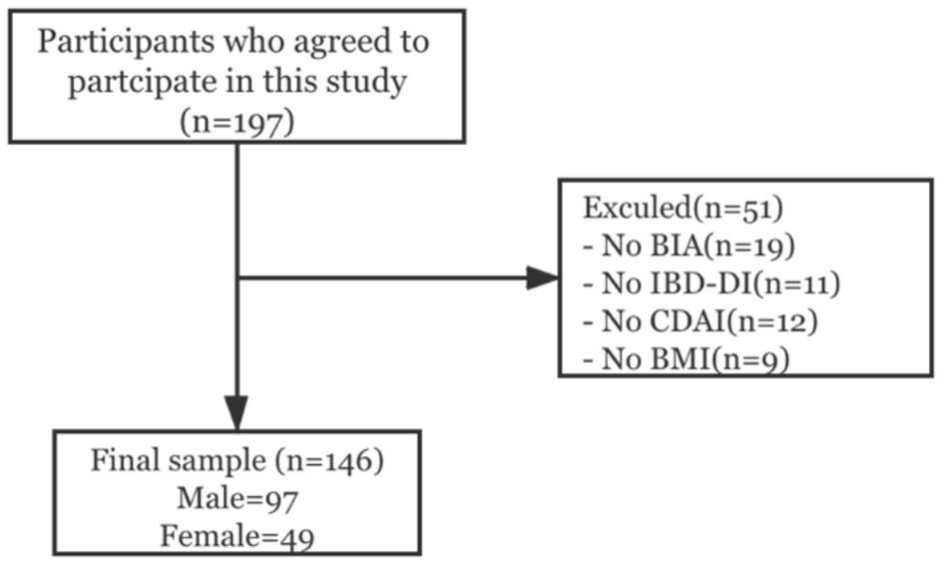
Flow chart of patients included in the study.

In this cross-sectional study, CD outpatients (*n* = 197) under anti-TNF-α therapy were included at Ruijin Hospital in Shanghai from February 2019 to June 2019. All CD patients were included in the study, independent of occupational activity, gender, race, or social class. Specifically, these participants were diagnosed with CD by their treating physicians and met the criteria established by the Inflammatory Bowel Disease Group of the Chinese Society of Gastroenterology. The following exclusion criteria were applied: patients with metabolic diseases (e.g., diabetes, hyperthyroidism) whose related indices were not well-controlled after hospitalization; patients with gastrointestinal tumors or tumors at other sites; patients with impaired water and mineral metabolism caused by cortical hormone therapy; and patients with metal implants (such as cardiac pacemakers) that could influence bioelectrical impedance results. Fifty-one patients were not included for missing values in the variables of body composition, BMI or others (Figure 1). The final sample was comprised of 146 participants.

The participants were informed of the study objective before the experiments started. Informed consent was obtained from each participant, who also consented to the publication of relevant data. This study was approved by the Ethics and Research Committee of Ruijin Hospital.

Before the start of the study, a pilot study consisting of 15 participants was conducted as a preliminary investigation and to pretest the survey instrument and subsequently adjust the scale. All researchers involved in this study were trained together. The samples were described using a structured questionnaire, including sociodemographic data and lifestyle.

The following information was collected: (a) sociodemographic data, including age, gender, height, weight, and smoking and drinking habits; (b) disease characteristics, including disease duration and disease activity assessed by Crohn's disease activity index (CDAI). Patients with CDAI ≤150 were considered in clinical remission, whereas those with CDAI >150 were considered in the active phase.

### Body Composition Measurement

We assessed body composition by multifrequency bioelectrical impedance analysis using the InBody S10 body water analyzer (InBody Korea). We also evaluated the total fat mass, body fat percentage, total muscle mass, skeletal muscle mass, and the muscle/fat ratio of the patients. This analyzer processes 30 impedance measurements by using six different frequencies (1, 5, 50, 250, 500, 1,000 kHz) in each of five segments of the body (right arm, left arm, trunk, right leg, and left leg) and 15 reactance measurements by using tetrapolar 8-point tactile electrodes at three different frequencies (5, 50, and 250 kHz) in each of the aforementioned segments.

The appendicular skeletal muscle mass (ASM) was measured as the sum of the muscle mass from the four limbs, and the ASM index (kg/m^2^) was calculated. Loss of skeletal muscle mass was determined based on the criteria set by the Asian Working Group for Sarcopenia (2019) (21): ASMI <7.0 kg/m^2^ for men and <5.7 kg/m^2^ for women.

### Handgrip Strength Evaluation

Measurement was performed with the participant in the following position: seated on an armless chair, with feet supported on the floor, hips and knees flexed at 90°, arms parallel to the body, elbows flexed at 90°, and forearms and wrists in a neutral position. The dominant side was measured three times at 1-min intervals, with verbal stimulation applied. The results were expressed in kilogram-force (kg), and the mean of the three measures was used. Muscle weakness was determined based on the criteria set by the Asian Working Group for Sarcopenia (21): handgrip strength <28 kg for men, and <18 kg for women.

### Nutritional Assessment

Patient-Generated Subjective Global Assessment (PG-SGA) was recommended as a nutritional assessment scale for IBD patients by the Inflammatory Bowel Disease Group of the Chinese Society of Gastroenterology. The PG-SGA assessment included (i) body weight, (ii) dietary intake, (iii) symptoms, (iv) movement and body function, (v) relationship between disease and nutritional requirement, (vi) metabolism requirement, and (vii) physical examination. According to the criteria, the scores indicated the following: 0–1, good nutritional status; 2–3, light malnutrition requiring nutritional education and/or nutritional intervention; 4–8, moderate malnutrition requiring nutritional intervention; and ≥9, severe malnutrition requiring symptom improvement and nutritional therapy. Patients with scores ≥4 were defined as having moderate or severe malnutrition. Those with scores <4 were enrolled in the low PG-SGA group.

### Inflammatory Bowel Disease Disability Index (IBD-DI)

CD-related disability was assessed based on the IBD-DI (13). The original IBD-DI comprises 19 items capturing five aspects of the ICF categories, including general health, body function (sleep/energy, affect, body image, pain, diarrhea, body mass index, and weight loss), body structure (presence of blood in the stool, arthralgia/arthritis), participation activity (regulating defecation, looking after own health, interpersonal activities, and work/education), and environmental factors (exacerbating effect or medication, food, family, and health care professional). A simplified version of the IBD-DI, which contains 14 items, was validated in 2017. All items were rescaled to range from 0 to 4. Binary items were coded as “0” for “no” and “4” for “yes.” BMI > 18.5 was assigned a score of “0,” whereas BMI < 18.5 was assigned a score of “4.” The total score ranged from 0 to 100 using the following formula: score × 100/(p × 4), where p represents the number of answered items. The total scores and their corresponding classifications were as follows: 0–20 (no disability), 20–35 (mild disability), 35–50 (moderate disability), and 50–100 (severe disability).

### Statistical Analysis

The data were analyzed using SPSS version 22.0. *P* < 0.05 indicated statistical significance. For normally distributed continuous data, the mean (standard deviation [SD]) was used as the measure of central location, whereas for continuous data with non-normal distribution, the median (interquartile range) was used. These characteristics were compared between the moderate to severe disability group and the no disability and minimal disability group by using Pearson's chi-square or Fisher's exact test to compare proportions. The odds ratio (OR) and its 95% confidence interval (CI) were calculated to determine the association of risk factors with moderate or severe disabilities. Statistically significant variables in the univariate analyses were then included in a multivariate regression model to identify the independent risk factors of moderate to severe disability by backward elimination analysis.

## Results

### Patient Characteristics

Table 1 presents the demographics and baseline characteristics of the population. All patients who completed the study allowed the use of their administrative data, which were thus included in this study. The mean age of the participants was 38.89 ± 10.68, 66.43% were male, 52.05% had moderate or severe disabilities, 28.76% had moderate or severe malnutrition (PG-SGA≥4), and 40.41% had sarcopenia. The average duration of IBD was 5.59 y. The disease was considered clinically in remission in 120 patients. The IBD disability index for all CD patients was 33.62 ± 9.63 (0–61).

**Table 1:** Characteristics of the study participants.

**Characteristics**	**Total (*n* = 146)**	***N* (%)**
	**Mean ± SD**	
Age, years	31.58 ± 10.00	
**Gender**		
Male		97 (66.43%)
Female		49 (33.56%)
BMI. Kg/m^2^	19.67 ± 3.37	
Smoking		12 (8.22%)
Drinking		1 (0.68%)
Duration of disease (years)	4.99 ± 4.91	
**IBD-DI**		
Moderate to severe disability		76 (52.05%)
Without or minimal disability		70 (47.95%)
**Malnutrition**		
PG-SGA≥4		42 (28.76%)
PG-SGA <4		104 (71.23%)
**Sarcopenia**		
Yes		41 (28.08%)
No		105 (71.92%)
**Disease activity**		
Active phase		26 (17.81%)
Remission phase		120 (82.19%)
High level CRP, ≥10 mg/L		29 (19.86%)
Hemoglobin, g/L	127.82 ± 19.60	
Albumin, g/L	41.10 ± 5.58	

Table 2 showed baseline characteristics of activity and remission phase participants of the CD patients. The active phase subjects had a higher CRP level (80.77 vs. 6.67%, *P* = 0.012), moderate to severe disability (92.31 vs. 43.33%, *P* < 0.001), PG-SGA≥4 (69.23 vs. 20.00%, *P* < 0.001) compared to the remission phase. The active phase subjects were also more frequently disability and sarcopenia compared with patients with the remission phase.

**Table 2:** Activity phase differences in the prevalence of sarcopenia and disability in CD patients.

	**Activity (*n* = 26)**	**Remission (*n =* 120)**	***P***
**Anthropometric indicators**			
Age, y	33.47 ± 10.09	31.72 ± 11.85	0.452
**Sex**			0.247
Male	14 (53.85%)	83 (69.17%)	
Female	12 (46.15%)	37 (30.83%)	
BMI, kg/m^2^	18.37 ± 2.58	19.96 ± 3.47	0.027^*^
**Biochemical indexes**			
High level CRP, ≥10 mg/L	21 (80.77%)	8 (6.67%)	0.012^*^
Hemoglobin, g/L	110.35 ± 22.41	131.60 ± 16.77	0.000^*^
Albumin, g/L	35.35 ± 4.39	42.54 ± 4.79	0.000^*^
**Body composition**			
BFM, kg	10.21 ± 5.83	9.27 ± 5.92	0.948
ASMI, kg/m^2^	6.39 ± 1.07	7.03 ± 1.13	0.010^*^
FFM, kg	44.71 ± 8.91	49.81 ± 9.73	0.083
TBW, kg	31.63 ± 5.86	34.18 ± 7.65	0.135
VFA, cm^2^	31.63 ± 29.50	36.58 ± 28.94	0.433
PBF, %	14.60 ± 7.40	16.42 ± 7.95	0.338
BCM, kg	28.90 ± 5.64	31.74 ± 7.03	0.087
Handgrip strength, kg	27.60 ± 8.27	32.32 ± 9.69	0.023^*^
**Nutritional assessment**			
PG-SGA≥4	18 (69.23%)	24 (20.00%)	0.000^*^
PG-SGA <4	8 (30.77%)	96 (80.00%)	
**IBD-DI**			
Moderate to severe disability	24 (92.31%)	52 (43.33%)	0.000^*^
Without to minimal disability	2 (7.69%)	68 (56.67%)	
**Sarcopenia**			
Yes	13 (50.00%)	28 (23.33%)	0.008^*^
No	13 (50.00%)	92 (76.67%)	

*Data are presented as mean ± standard deviation (median) or as stated*.

*BFM, body fat mass; VFA, visceral fat area; PBF, percent body fat; BCM, body cell mass; CRP, Creactive protein; BMI, body mass index; ASMI, appendicular skeletal muscle mass index; FFMI, fat free mass index; FFM, fat free mass; PG-SGA, Patient-Generated Subjective Global Assessment; CDAI, Crohn's disease activity index*.

*p-value from a t-test for independent samples or a chi-square test, depending on the type of variable*.

* *p < 0.05. Continuous variables (mean ± SD) and categorical variables (percentage)*.

### General Characteristics of CD Patients With Moderate to Severe Disability vs. Without or Minimal Disability

Table 3 lists the body composition, sociodemographic factors, age, nutritional-biochemical indexes, nutritional status of the patients by their disability status. The moderate to severe disability group had lower values for body weight, BMI, BFM, ASMI, and handgrip strength, compared with the group without to minimal disability (*P* < 0.05). The moderate to severe disability group had higher CDAI and PG-SGA scores, compared with the without to minimal disability group (*P* < 0.05). No significant differences in age, duration of disease, and hemoglobin levels were found between the moderate to severe disability group and the without to minimal disability group (*P* > 0.05). The prevalence of sarcopenia (48.68 vs. 31.43%, *P* = 0.043), PG-SGA≥4 (39.47 vs. 17.14%, *P* = 0.003), and high-level CRP (27.63 vs. 11.43%, *P* = 0.021) were higher in the moderate to severe disability group than in the without to minimal disability group.

**Table 3:** General characteristics of CD patients with moderate to severe disability vs. Without to minimal disability.

	**Moderate to severe disability (*n =* 76)**	**Without to disability (*n =* 70)**	***P***
**Anthropometric indicators**			
Age, y	34.27 ± 11.31	31.67 ± 10.05	0.236
Sex, female, (%)	36 (47.37%)	20 (28.57%)	0.027^*^
Height, cm	165.26 ± 8.91	170.89 ± 9.40	0.001^*^
Body weight, kg	51.67 ± 12.31	61.79 ± 12.84	0.000^*^
BMI, kg/m^2^	19.56 ± 3.22	21.68 ± 3.06	0.001^*^
Duration of disease, >5 y	29 (38.16%)	27 (38.57%)	0.547
CDAI index	115.58 ± 75.93	60.23 ± 48.21	0.000^*^
**Biochemical indexes**			
Hemoglobin, g/L	124.87 ± 20.54	131.01 ± 18.13	0.058
Albumin, g/L	40.07 ± 5.54	42.20 ± 5.45	0.021^*^
High-level CRP, ≥10 mg/L	21 (27.63%)	8 (11.43%)	0.021^*^
**Nutritional assessment**			
PG-SGA≥4	30 (39.47%)	12 (17.14%)	0.003^*^
PG-SGA <4	46 (60.52%)	58 (82.86%)	
**Body composition**			
ASMI, kg/m^2^	6.59 ± 1.07	7.27 ± 1.12	0.000^*^
FFM, kg/m^2^	43.29 ± 8.57	48.93 ± 10.79	0.001^*^
BFM, kg/m^2^	8.77 ± 5.59	11.46 ± 5.82	0.000^*^
VFA, cm^2^	28.88 ± 27.74	43.19 ± 28.69	0.003^*^
WC, cm	67.97 ± 8.68	74.21 ± 9.03	0.000^*^
AC, cm	25.02 ± 3.28	27.40 ± 3.71	0.000^*^
BCM, L	28.73 ± 6.11	33.57 ± 6.77	0.000^*^
TBW, kg	31.84 ± 6.41	35.76 ± 7.85	0.002^*^
PBF, %	14.95 ± 6.96	17.16 ± 8.53	0.126
Handgrip strength, kg	28.61 ± 9.30	34.61 ± 8.96	0.000^*^
**Sarcopenia**			
Yes	37 (48.68%)	22 (31.43%)	0.043^*^
No	39 (51.32%)	48 (68.57%)	

*Data are presented as mean ± standard deviation (median) or as stated*.

*CRP, C-reactive protein; BMI, body mass index; ASMI, appendicular skeletal muscle mass index; FFMI, fat-free mass index; FFM, fat-free mass; BFM, body fat mass; PG-SGA, Patient-Generated Subjective Global Assessment; VFA, visceral-fat area; WC, waist circumference; AC, arm circumference; CDAI, Crohn's disease activity index*.

*p-value from a t-test for independent samples or a chi-square test, depending on the type of variable*.

* *p < 0.05. Continuous variables (mean ± SD) and categorical variables (percentage)*.

### Correlations Between the IBD-DI and Clinical Variables

**Figure 2:**
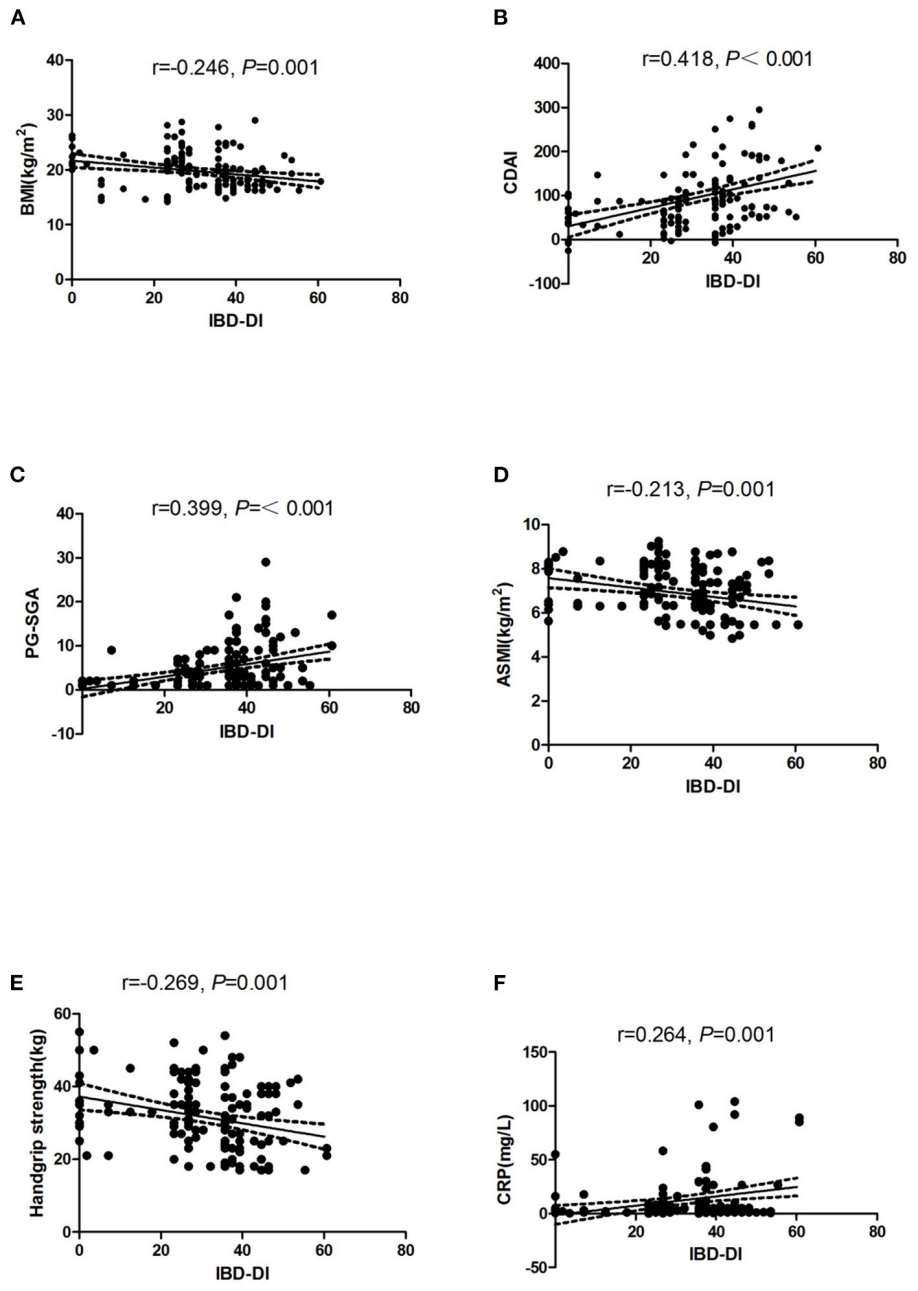
Dispersion graphs depicting correlations between IBD-DI and BMI **(A)**, CDAI **(B)**, PG-SGA **(C)**, ASMI **(D)**, Handgrip strength **(E)**, and CRP **(F)**. *P*-values on each graph were calculated for all CD patients. r indicates the Spearman's rank correlation coefficient. IBD-DI, inflammatory bowel disease disability index; BMI, body mass index; CDAI, Crohn's disease activity index; PG-SGA, Patient-Generated Subjective Global Assessment; ASMI, appendicular skeletal muscle mass index; CRP, C-reactive protein.

In CD patients, the variables that were significantly correlated with the IBD-DI were BMI, CDAI, PG-SGA, ASMI, handgrip strength, and CRP (Figure 2).

### Risk Factors Associated With Moderate to Severe Disability

In univariate regression models, disability was significantly associated with BMI (OR: 4.60, 95% CI: 2.28–9.30), sex (OR: 2.25, 95% CI: 1.13–4.47), CDAI (OR: 15.69, 95% CI: 3.54–69.42), BMI (OR: 4.60, 95% CI: 2.28–9.30), sarcopenia (OR: 3.40, 95% CI: 1.59–7.72), and CRP (OR:1.31, 95% CI: 1.05–1.64) (Table 4).

**Table 4:** Risk factors associated with moderate to severe disability for all CD patients.

**Variables**	**Univariate analysis**		**Multivariate analysis**	
	**OR (95%CI)**	***p***	**OR (95%CI)**	***p***
**Sex**				
Male	1		1	
Female	2.25 (1.13–4.47)	0.021^*^	1.64 (0.70–3.82)	0.254
**Duration of disease**				
<5years	1			
≥5years	0.98 (0.50–1.91)	0.959		
**Disease activity**				
Remission	1		1	
Active	15.69 (3.54–69.42)	<0.001^*^	10.47 (2.09–52.42)	0.004^*^
**BMI**				
≥18.5 kg/m^2^	1		1	
<18.5 kg/m^2^	4.60 (2.28–9.30)	<0.001^*^	4.11 (1.80–9.38)	0.001^*^
**Sarcopenia**				
No	1		1	
Yes	3.40 (1.59–7.72)	0.002^*^	1.29 (0.47–3.53)	0.620
**Malnutrition**				
No	1		1	
Yes	3.15 (1.46 to 6.83)	0.004^*^	1.79 (0.69 to 4.66)	0.233
**CRP**				
≥10 mg/L	1		1	
<10 mg/L	1.31 (1.05 to 1.64)	0.017^*^	1.11 (0.85 to 1.45)	0.444
**Albumin**				
≥35 g/L	1		1	
<35 g/L	2.44 (0.94 to 6.35)	0.065	1.39 (0.41 to 4.74)	0.604

*Data are presented as mean ± standard deviation (median) or as stated*.

*Univariate analysis: binary logistic regression analysis unadjusted; Multivariate analysis: After univariate analysis, all variables with P <0.15 were considered in subsequent multivariate analyses*.

*CRP, C-reactive protein; BMI, body mass index; PG-SGA, Patient-Generated Subjective Global Assessment; CDAI, Crohn's disease activity index; Malnutrition, PG-SGA≥4*.

* *p < 0.05*.

By multivariate analysis, the BMI (OR:4.11, 95% CI: 1.80–9.38) and disease activity (OR:10.47, 95% CI: 2.09–52.42) were identified as the most significant factors predicting disability.

## Discussion

In the past 20 years, CD has markedly increased and become a common disease of the digestive system in China (22). About 70–80% of CD patients may suffer from intestinal obstruction, abdominal abscess, intestinal perforation, and other complications. CD patients may receive surgery, high medical costs. A complicated disease course increases disability, including serious damage to the physical and mental health of patients, a decline in the quality of life (23), and work efficiency (24–26), and even depression (27). In the current study, we confirmed CD in the active phase to be significantly associated with disability.

We used the scale developed by Gower-Rousseau et al., which consisted of 14 items. The later Korean, Portuguese, and Prevost versions containing 14 items showed good reliability and validity. We determined the average IBD-DI score, which was 33.62 ± 9.63, similar results were observed in the Gower-Rousseau study. In the current study, 52.05% of the CD patients had a moderate to severe disability, whose results were similar to those reported by Marinelli et al. (28) and Yoon et al. (23). Notably, the results of the multivariate analysis indicated that disease activity was an independent risk factor for disability in patients with CD (OR:10.47, 95%CI:2.09–52.42). In a validation study from Australia (29), disability was significantly correlated with CDAI, and lBD-Q. A study among Koreans showed that disability in patients with IBD was correlated with disease activity and poor quality of life (23, 29). A meta-analysis found a significant correlation between disease activity and disability in IBD patients (30). All of these observations confirmed that disease activity is an important factor affecting disability. Therefore, effective treatment measures should be applied to reduce the degree of disease activity in patients.

BMI was another independent predictor for CD patients with a moderate to severe disability in this study (OR:4.11, 95 CI: 1.80–9.38). The patients with moderate to severe disability had lower BMI than those without or with minimal disability (19.56 ± 3.22 vs. 21.68 ± 3.06, *P* = 0.001). However, this was the first study to concurrently demonstrate that BMI was significantly associated with disability. A Portugal study reported that BMI was related to the quality of life in IBD patients; moreover, the effect of BMI on the psychological and physical quality of life was mediated via the mechanisms of body image (31). An Israel study enrolled 100 IBD patients and found that a lower BMI was associated with a more severe disease course (32). A case-control study revealed that women with ulcerative colitis exhibited decreased lower limb strength and mobility limitations, which were associated with BMI (33). Besideds, a systematic review indicated that BMI was normally lower in CD patients, and regular medical therapy could not improve BMI in these patients (34). Our previous study (35) demonstrated that the dietary structure of IBD patients was unreasonable, characterized by insufficient intake of energy and protein; in addition, lack of physical activity can lead to body muscle depletion. Wardle et al. (36) found an increased prevalence of disordered eating behavior in CD and a greater prevalence of binge eating, food craving, low mood, and high anxiety. Moreover, Chan et al. (27) reported that symptoms of anxiety and depression were independently associated with IBD-related disability. Arigo et al. (37) suggested that fear and anxiety surrounding gastrointestinal symptoms can lead to disordered eating practices of a restrictive nature. In a French survey (38), nearly half of the subjects reported that the disease had changed the pleasure of eating, with only a quarter of the patients eating a normal diet during relapse. Thus, reduced dietary intake and disordered eating behavior can potentially lower BMI and aggravate disability which may associated with anxiety and depression.

Studies on the association between IBD-related disability and muscle-related sarcopenia have rarely, if ever, been reported. A previous study determined that the prevalence of sarcopenia among adult Chinese patients with CD was 60% (39). In the current study, the prevalence rates of sarcopenia were lower than the previously reported rate in Chinese patients. The discrepancies were most likely attributable to significant differences in patient selection and the methods used. We found that the muscle mass in remission was significantly higher than that in activity, which is consistent with our previous findings. Notably, patients with moderate to severe disability had lower sarcopenia-related index than patients with without to minimal disability (ASMI: 6.91 ± 1.12 vs. 7.59 ± 0.97, *P* = 0.002, handgrip strength: 28.61 ± 9.30 vs. 34.61 ± 8.96, *p* < 0.001). Research has shown that sarcopenia is a progressive and generalized syndrome characterized by the loss of skeletal muscle mass and muscle strength with adverse outcomes, such as frailty, poor quality of life, and mortality (40, 41). The pathogenesis of muscle wasting includes several elements, such as aging, systemic inflammation, mitochondrial dysfunction, increased proteolysis, decreased proteosynthesis, and insulin resistance (40). Cravo et al. reported that reduced lower muscle attenuation seemed to be associated with more severe phenotypes in patients with CD (42). These conditions may aggravate disability in CD patients. Univariate analysis showed that moderate to severe disability was significantly associated with CD-related sarcopenia; however, multivariate analysis revealed no such finding.

In the univariate analysis, moderate to severe disability was found in 2.25 times more female than male CD patients. This finding is consistent with the studies conducted among French (13) and Spanish (14) subjects, which also found a higher IBD-DI in female than male subjects. By contrast, the current study did not find the same result after multivariate analysis. Similarly, a Dutch study observed no link between disability and sex (43). In addition, the data indicated that patients in the moderate to severe disability group had higher CRP levels than those in the without to minimal disability group (27.63 vs. 11.43%, *P* = 0.021). However, this finding was not supported by the multivariate analysis. Nonetheless, close attention to the clinical CRP score should be given attention.

In China, CD patients have poor access to IBD treatment centers and specialist doctors. Moreover, doctors focus more on disease treatment and remission than the quality of life and disability in CD patients (44). Although an increasing number of studies have reported that disability in CD can affect their work efficiency and psychological status, reports on Chinese-related populations were limited. Thus, CD patients in China must monitor the conditions of their disability during clinical treatment. Moreover, prompt corrective measures have to be undertaken to alleviate body mass depletion and maintain remission, consequently improving the quality of life and reducing disability. A multidisciplinary assessment of patients with CD is always encouraged, and nutrition strategies should always be suitable to the needs of the patient.

We also identified several limitations. First, this study is a single-center cross-sectional study. This research sample size was small and the size of the cohort was only defined by the number of consecutive outpatients during the sample collection. Selection bias could not be excluded and not all Chinese CD patients could be represented, considering the sample; moreover, the correlation between patient disability and demographic and disease characteristics at a certain time node does not imply causality. Second, selection bias could not be avoided because not all patients who were surveyed were willing to participate. Third, a cross-sectional protocol provides limited evidence concerning changes over time, which would be better assessed by a future longitudinal study. Furthermore, multicenter, larger sample clinical observational research, longitudinal follow-up and intervention studies would be required to determine the reversal of disability for the successful management of disease activity and BMI.

## Conclusions

We demonstrated that disability is strongly related to disease activity and body mass in CD patients. In the management of the disease, not only the regular progression of the disease but also disability and BMI should be investigated during the regular treatment of clinical outpatients. In conclusion, close monitoring and follow-up should be conducted for patients with a high risk of disability, and effective measures should be adopted, which may be the best approach to preventing disability and helping patients return to normal life. This would also allow for a novel multidisciplinary approach based on collaborative efforts between gastroenterologists, nurses, nutritionists, and psychologists to improve the quality of life in general.

## Data Availability Statement

The original contributions presented in the study are included in the article/supplementary material, further inquiries can be directed to the corresponding author/s.

## Ethics Statement

The studies involving human participants were reviewed and approved by Ethics and Research Committee of Ruijin Hospital. The patients/participants provided their written informed consent to participate in this study.

## Author Contributions

DB, JZ, and YS: conceptualization. DB, YG, YJ, ZH, and YT: investigation. DB, JZ, and YS: methodology. YS: funding acquisition. DB and YJ: data curation. DB: writing—original draft preparation. YG, ZH, JZ, and YS: writing—review and editing. YT, JZ, and YS: project administration. JZ and YS: supervision. QC and ZH: validation. All authors have read and agreed to the published version of the manuscript.

## Conflict of Interest

The authors declare that the research was conducted in the absence of any commercial or financial relationships that could be construed as a potential conflict of interest.
